# Efficacy and Safety of Everolimus for Maintenance Immunosuppression of Kidney Transplantation: A Meta-Analysis of Randomized Controlled Trials

**DOI:** 10.1371/journal.pone.0170246

**Published:** 2017-01-20

**Authors:** Jinyu Liu, Dong Liu, Juan Li, Lan Zhu, Chengliang Zhang, Kai Lei, Qiling Xu, Ruxu You

**Affiliations:** 1 Department of Pharmacy, Tongji Hospital, Tongji Medical College, Huazhong University of Science and Technology, Wuhan, Hubei, China; 2 Institute of Organ Transplantation, Tongji Hospital, Tongji Medical College, Huazhong University of Science and Technology, Wuhan, Hubei, China; 3 Department of Biotechnology and Molecules, Assumption College, Worcester, Massachusetts, United States of America; 4 Department of Pharmacy, Union Hospital, Tongji Medical College, Huazhong University of Science and Technology, Wuhan, Hubei, China; University of Toledo, UNITED STATES

## Abstract

**Background:**

Conversion to everolimus is often used in kidney transplantation to overcome calcineurin inhibitor (CNI) nephrotoxicity but there is conflicting evidence for this approach.

**Objectives:**

To investigate the benefits and harm from randomized clinical trials (RCTs) involving the conversion from CNI to everolimus after kidney transplantation.

**Methods:**

Databases were searched up to March 2016. Two reviewers independently assessed trials for eligibility and quality, and extracted data. Results are expressed as risk ratio (RR) or mean difference (MD) with 95% confidence intervals (CI).

**Results:**

Eleven RCTs, with a total of 1,633 patients, met the final inclusion criteria. Patients converted to everolimus had improved renal function at 1 year posttransplant with an estimated glomerular filtration rate (eGFR) of 5.36 mL/min per 1.73 m^2^ greater than patients remaining on CNI (p = 0.0005) and the longer-term results (> 1 year) of renal function was identical to that of 1 year. There was not a substantial difference in graft loss, mortality, and the occurrence of adverse events (AEs) or serious AEs. However, the risks of acute rejection and trial termination due to AEs with everolimus are respectively 1.82 and 2.63 times greater than patients staying on CNI at 1 year posttransplant (p = 0.02, p = 0.03, respectively). Further, those patients who converted to everolimus had a substantially greater risk of anemia, hyperlipidemia, hypercholesterolemia, hypokalemia, proteinuria, stomatitis, mouth ulceration, and acne.

**Conclusions:**

Conversion from CNI to everolimus after kidney transplantation is associated with improved renal function in the first 5 years posttransplant but increases the risk of acute rejection at 1 year posttransplant and may not be well endured.

## Introduction

Kidney transplantation is the treatment of choice for most patients with end-stage renal disease. Strategies to increase donor organ availability and to prolong the transplanted kidney’s survival have become priorities in kidney transplantation. Immunosuppressive therapy is essential and significant in this respect, nevertheless, but choosing the best suitable immunosuppressive therapy is still fairly complex. The calcineurin inhibitors (CNIs) are the principal components of immunosuppressive therapy after kidney transplantation and have made a major contribution to current long-term transplant outcomes[1, 2]. Meanwhile, tacrolimus has been recommended as a first-line agent for kidney transplantation recipients in kidney disease improving global outcomes (KDIGO) in 2009 [3]. However, CNIs are associated with a number of potentially serious side effects, including nephrotoxicity, diabetes, hypertension, and neurotoxicity that contribute to morbidity and mortality after transplantation [4–8]. Upon the fifth year, 90% of grafts revealed evidence of CNI-related lesions, and whereas early acute nephrotoxicity is typically reversible with CNI minimization; chronic lesions cannot be altered once initiated [5]. Furthermore, combined therapy with tacrolimus and mycophenolic acid may be associated with a particularly higher risk of BK infection [9], which is the significant and dangerous factor in the failure of the transplanted kidney. Therefore, diminishing or even eliminating CNI has become the focus of further optimization of immunosuppressive therapy in renal transplantation.

Everolimus (EVR), a mammalian target of the rapamycin inhibitor (mTORi), works 100 times greater than ciclosporin. It is part of a distinguished group of immunosuppressive drugs that have a divergent mode of action to that of CNIs even though they bind to the identical intracellular immunophilin as tacrolimus, namely, FKBP12. The mTORi/FKBP12 complex binds to and hinders the TORC1 complex, preventing proliferation of numerous cell types, such as T-lymphocytes, alloprimed B cells, and CD8 TAb-supp cells [10, 11]. The side effect profile of EVR is different from that of CNI and includes impaired wound healing, mouth ulcers, stomatitis, arthralgia, hyperlipidemia, and anemia [12–15]. The KDIGO instructions suggest that EVR should not be initiated prior to surgical wounds healing [3]. However, EVR may improve renal function and reduce the occurrence of malignancy, which makes it an attractive alternative to CNIs for maintenance therapy after kidney transplantation [16, 17], even though it does cause glomerular disease in some patients resulting in marked proteinuria [18]. A number of randomized controlled trials (RCTs) have examined the potential benefits of introducing EVR after kidney transplantation using a variety of protocols that differ with respect to the timing and mode of conversion to EVR, whether CNIs are eliminated or diminished, and in the level of baseline renal function at the time of EVR introduction. Such studies have provided conflicting results on the efficacy and side effect profile of EVR.

Up to now, there is no published work that systematically reviews EVR used as a maintenance therapy in renal transplant recipients. We undertook the meta-analysis of randomized controlled trials (RCTs) to systematically identify and summarize the current available evidence of the short- and long-term benefits and harm of conversion from CNI to EVR-based maintenance immunosuppression for kidney transplant recipients.

## Materials and Methods

### Information Sources and Search Strategies

A systematic literature search was performed using Pubmed/MEDLINE, OVID/EMBASE, and Cochrane Library covering the period from the inception of the database until March 2016 using a predefined algorithm (S1 Table) without language restrictions. References included in pertinent systematic reviews were also screened.

### Eligibility Criteria

Every RCT examining the conversion from CNI to EVR-based maintenance immunosuppression in adult isolated kidney transplantation was assessed. Research was judged to be suitable if they examined sudden or slow conversion to EVR, in the initial or later kidney transplant recipients, regardless of the time posttransplantation and baseline renal function. Trials that were deemed suitable included those in which the intervention (conversion to EVR) and reference (CNI continuation) groups were administered more maintenance immunosuppression comprising antimetabolites (mycophenolate or azathioprine) and steroids. Observational and uncontrolled studies, those examining children, those in which the participants obtained other solid organs plus a renal transplantation, and animal trials were eliminated.

### Data Extraction

Data removal was done separately by 2 investigators (Jinyu Liu and Ruxu You) and discrepancies were resolved by a third investigator (Juan Li). We documented data on trial characteristics and demographics, such as authors’ names, year published, journal’s name, sample size per-arm, population characteristics, indications, CNI dose, EVR characteristics (bolus and maintenance dose, mode of administration, timing of intervention, sudden stopping or tapering of prior immunosuppression) and length of the trial. The main meta-analysis result was renal function (estimated Glomerular Filtration Rate, eGFR) and secondary results were acute rejection, graft loss, patient survival and AEs. All of the results were investigated 1 year posttransplantation; long-term results, if available, were also investigated.

### Assessment of Methodological Quality

The methodological quality of the included trials and the risk of bias were evaluated with elements from the Cochrane Collaboration’s tool for determining the risk of bias [19, 20]. The domains implemented in the current systematic review concerned randomization and allocation concealment (selection bias), blinding (performance and detection bias), loss to follow-up, and keeping to the intention-to-treat principle (attrition bias), selective reporting (reporting bias), and other biases. We decided to put forth the meta-analysis of every one of the studies while offering a synopsis of the risk of bias across trials.

### Data Synthesis and Statistical Analysis

For every study we recovered, we computed the crude risk ratio (RR) estimates and corresponding 95% confidence intervals (CI) for the evaluated binary results (acute rejection, graft loss, patient survival, and AEs), and the mean difference (MD) and corresponding standard deviation (SD) for the evaluated continuous outcomes (GFR). The existence of statistically significant heterogeneity was evaluated using the Cochran Q test and the degree of the observed heterogeneity was evaluated by the I^2^ (ranging from 0% to 100%). A Cochran’s Q p < 0.10 was considered to show significant heterogeneity, and analysis was undertaken using the random effects model. Otherwise, the fixed effects model was used. Summary effect estimates for renal function, acute rejection, graft loss, and patient survival were set on intention-to-treat (ITT) groups as identified in the study publications; further, all qualified trials documented AEs in the safety population and, therefore, summary effect sizes for AEs were approximated using safety populations as stated in the study publications.

Pre-determined subgroup analyses were done to investigate origins of heterogeneity; trials were stratified by time of conversion to EVR (early versus late conversion, defined as ≤ 3 months after transplantation) or by the duration to follow up. Sensitivity analyses were done based on to the protocol examining GFR or acute rejection. To additionally determine moderators of the noted effect estimates, a meta-regression analysis was performed to account for baseline renal function. Publication bias was investigated using funnel plots. Where information was missing we contacted the researchers to seek the pertinent information. Analyses were done in RevMan 5.3 (Cochrane Collaboration, 2014) and STATA 10 (STATA Corp., College Station, TX). All p values were 2-tailed. The study is documented based on the favored reporting items for systematic reviews and meta-analyses checklist [21].

## Results

### Description of Studies

**Fig 1:**
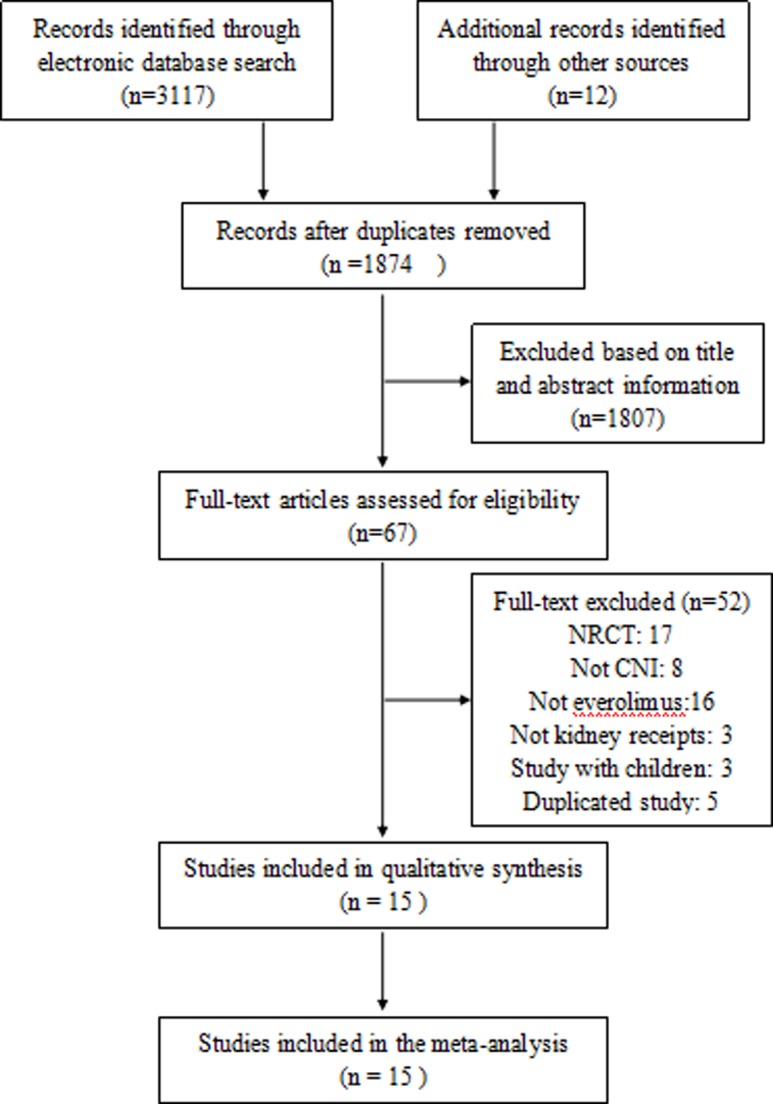
Flow chart of included studies in the meta-analysis.

We identified 3,129 possibly relevant references (PubMed, 673; Ovid, 1801; Cochrane Library, 643; and other sources, 12). After examining the titles and abstracts and eliminating identical publications, 67 possibly qualified articles were determined. Fifteen reports [22–36] of 11 trials, including a total of 1,633 randomized patients, were chosen for inclusion in the meta-analysis (Fig 1). One of these trials was available in abstract form only by Wolfgang et al 2012 [31]. Nine trials compared EVR to cyclosporine, and 2 trials compared EVR to CNI (cyclosporine or tacrolimus).

All of the incorporated studies were designed to determine the safety and efficacy of conversion from CNI to EVR in adult kidney transplant patients. Design characteristics, immunosuppression regimens, and reported results for every study are summarized in Table 1. The median sample size was 96 participants (interquartile range, 13–337), and the median treatment duration was 19.5 months (with a minimum of 6 months and a maximum of 60 months). All of the trials stated renal function, acute rejection, graft loss, patient survival, and AEs. GFR was estimated by Nankivell formula in 5 trails [24–27, 29–33], modification of diet in renal disease (MDRD) formula in 3 trails [22, 34, 35], and was unclear in 3 studies [23, 28, 36]. Early conversion to EVR (defined as ≤3 months after transplantation) was evaluated in 5 studies [22, 23, 25, 28, 30, 31], whereas 6 studies evaluated the late conversion to EVR [24, 26, 27, 31, 32–36]. There was a difference in baseline renal function, both within and between the trials, but most of the patients had mild or moderate renal dysfunction at the time of randomization.

**Table 1:** Characteristics of included studies.

Study ID	Study design	Time to conversion since transplant	Immunosuppressive dosing regime					Outcomes reported					follow up (m)
			Method of switching	Pre-conversion	Intervention	Comparator	CNI in the interventionarm after switching	GFR	Graft loss	Mortality	Acute rejection	AEs	
Wolfgang 2012 [31]	a prospective open-label multicenter RCT (N = 337)	3 m	Unclear	CsA, EC-MPS, steroids	EVR: Target level (5–10ng/ml), EC-MPS, steroids (N = 171)	CsA, EC-MPS, steroids (N = 166)	Unclear	√	√	√	√	√	12
Thibault 2016 [22]	a prospective multicenter open-label RCT (N = 13)	within 24 h	No conversion	No	EVR: Bolus (6mg/d), Target level (6–10ng/ml), EC-MPS, steroids (N = 7)	CsA, EC-MPS, steroids (N = 6)	None	√	√	√	√	√	6
Seckinger 2008 [35]	a prospective single-center RCT (N = 39)	6 m	Tapered	CsA, MPA, steroids	EVR: Target level (6–10ng/ml), MPA, steroids (N = 20)	CsA, MPA, steroids (N = 19)	CsA	√	√	√	√	√	15
Budde 2011 [33]	a prospective multicentrer parallel-group RCT (N = 300)	4.5 m	Tapered	CsA, EC-MP, steroids	EVR: Bolus (1.5mg/d), Target level (6–10ng/ml), EC-MPS, steroids (N = 154)	CsA, EC-MPS, steroids (N = 146)	CsA	√	√	√	√	√	12
Mjörnstedt 2012 [30]	an open-label multicenter RCT (N = 202)	7 w	Abrupt	CsA, EC-MP, steroids	EVR: Bolus (3mg/d), Target level (6–10ng/ml), EC-MPS, steroids (N = 102)	CsA, EC-MPS, steroids (N = 100)	None	√	√	√	√	√	12
Rostaling 2015 [23]	a prospective open-label multicenter RCT (N = 194)	3 m	Tapered	CsA, EC-MPS, steroids	EVR: Bolus (3mg/d), Target level (6–10ng/ml), EC-MPS, steroids (N = 96)	CsA, EC-MPS, steroids (N = 98)	CsA	√	√	√	√	√	12
Chadban 2014 [28]	a prospective multinational open-label RCT (N = 96)	2 w	Tapered	CsA, MPA, steroids	EVR: Target level (8–12ng/ml), steroids (N = 48)	CsA, MPA, steroids (N = 47)	CsA	√	√	√	√	√	12
Budde 2015 [27]	an open-label prospective parallel-group RCT (N = 93)	> 6m	Tapered	CsA or TaC, EC-MPS, steroids	EVR: Bolus (1.5mg/d), Target level (6–10ng/ml), EC-MPS, steroids (N = 46)	CsA or TaC, EC-MPS, steroids (N = 47)	CsA or TaC	√	√	√	√	√	12
Bemelman 2009 [34]	a prospective multicenter open-label RCT (N = 77)	6 m	Tapered	CsA, MPS, steroids	EVR: Target AUC12(150 mg·h/L), EC-MPS, steroids (N = 38)	CsA, MPS, steroids (N = 39)	CsA	√	√	√	√	√	24
Holdaas 2011 [32]	an open-label multicenter RCT (N = 250)	6 m	Tapered	CsA or TaC, MPA, Aza, steroids	EVR: Target level (6–10ng/mL), MPA, Aza, steroids (N = 127)	CsA or TaC, MPA, Aza, steroids (N = 123)	CsA or TaC	√	√	√	√	√	24
Kihm 2009 [36]	a prospective single-center RCT (N = 32)	12 m	Tapered	CsA, MPA, steroids	EVR: Bolus (3mg/d), Target level (6–10ng/ml), MPA, steroids (N = 17)	CsA, MPA, steroids (N = 15)	CsA	√	√	√	√	√	24
Budde 2012 [29]	a prospective multicentrer parallel-group RCT (N = 300)	4.5 m	Tapered	CsA, EC-MPS, steroids	EVR: Bolus(1.5mg/d), Target level (6–10ng/ml), EC-MPS, steroids (N = 154)	CsA, EC-MPS, steroids (N = 146)	CsA	√	√	√	√	√	36
Mjörnstedt 2015 [25]	an open-label multicenter RCT (N = 202)	7 w	Abrupt	CsA, EC-MPS, steroids	EVR:Bolus(3mg/d), Target level (6–10ng/ml), EC-MP, steroids (N = 102)	CsA, EC-MPS, steroids (N = 100)	None	√	√	√	√	√	36
Budde (ZEUS) 2015 [24]	a prospective multicentrer parallel-group RCT (N = 300)	4.5 m	Tapered	CsA, EC-MPS, steroids	EVR: Bolus (1.5mg/d), Target level (6–10ng/ml), EC-MPS, steroids (N = 154)	CsAEC-MPS steroids (N = 146)	CsA	√	√	√	√	√	60
Budde (APOLLO)2015 [26]	an open-label prospective parallel-group RCT (N = 93)	>6 m	Tapered	CsA or TaC, EC-MPS, steroids	EVR: Bolus (1.5mg/d), Target level (6–10ng/ml), EC-MPS, steroids (N = 46)	CsA or TaC, EC-MPS, steroids (N = 47)	CsA or TaC	√	√	√	√	√	60

CsA, cyclosporine A; EVR, everolimus; TaC, tacrolimus; Aza, azathioprine; MPA, mycophenolic acid; CNI,calcineurin inhibitor; EC-MPS, enteric-coated mycophenolate sodium; GFR, glomerular filtration rate; AEs, adverse effects; RCT, randomized controlled trail; h, hour; m, month; w, week.

### Risk of Bias in Included Studies

**Fig 2:**
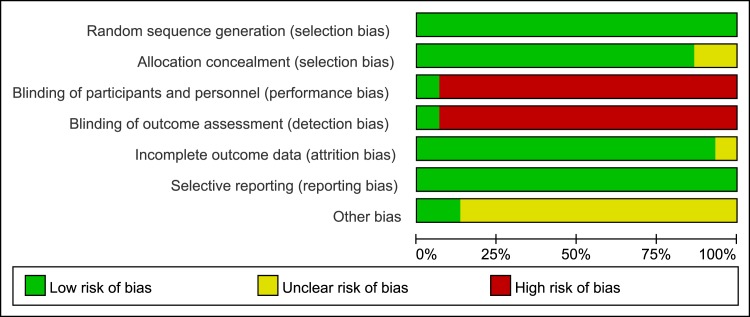
Quality of included studies in the meta-analysis.

The Cochrane Collaboration tool was implemented to evaluate the risk of bias (Fig 2 and S1 Fig). All trials were randomized, but 3 trials gave no indication of the allocation method used. Ten trials were open-label and only 1 trial reported blinding investigators [35]. Attrition was adequately reported in 10 trials and was generally low (< 20%). Intention-to-treat analysis was confirmed for 6 trials and was unclear for the remaining 5 trials.

### Assessed Outcomes and Evidence Synthesis

#### Renal function

**Fig 3:**
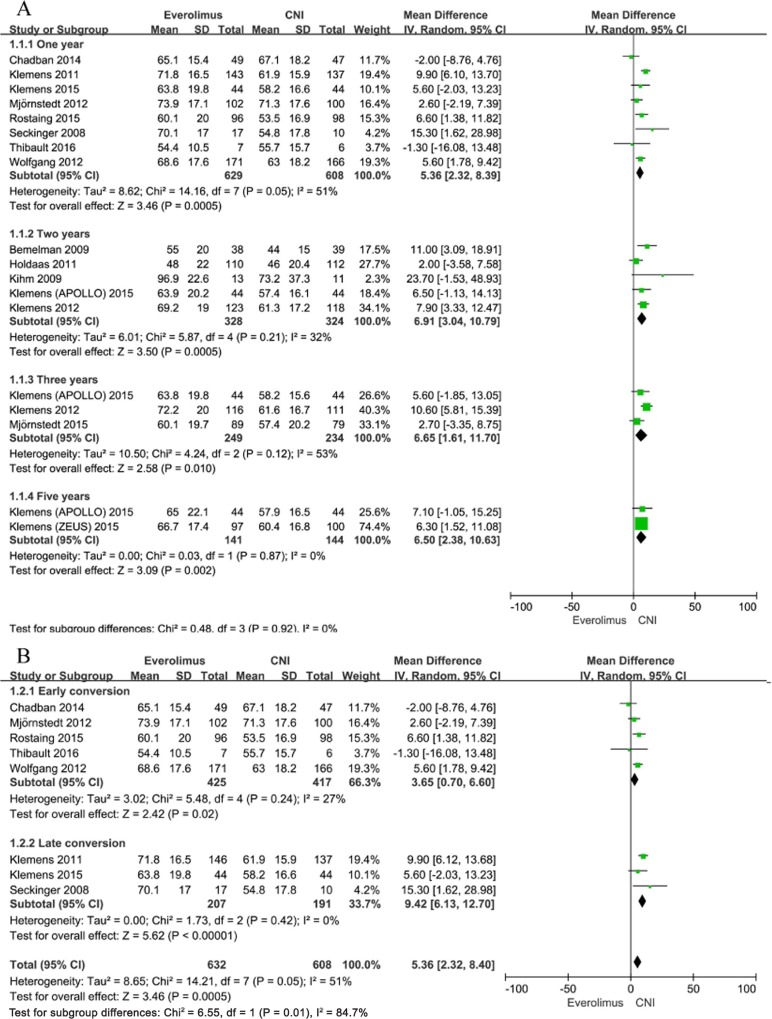
A. Everolimus versus calcineurin inhibitor; mean GFR up to 1–5 years after transplantation stratified by study duration. B. Everolimus versus calcineurin inhibitor; mean GFR up to 1 year after transplantation stratified by time to conversion.

Renal function was reported at 1, 2, 3, and 5 years after transplantation by 8, 5, 3 and 2 included studies, respectively. In the ITT analysis, patients converted to EVR had significantly better renal function at 1, 2, 3, and 5 years after transplantation compared to patients remaining on CNI [(MD, 5.36; 95% CI, 2.32–8.39; I^2^, 51%), (MD, 6.91; 95% CI, 3.04–10.79; I^2^, 32%), (MD, 6.65; 95% CI, 1.61–11.70; I^2^, 53%), and (MD, 6.50; 95% CI, 2.38–10.63; I^2^, 0%); Fig 3A]. When studies were stratified according to the time of conversion to EVR (early versus late conversion, defined as ≤ 3 months after transplantation), there was a statistically significant trend toward a more favorable GFR difference between EVR and CNI groups in the late conversion trials (MD, 9.42; 95% CI, 6.13–12.70; I^2^, 0%) compared to the early conversion trials (MD, 3.65; 95% CI, 0.70–6.60; I^2^, 27%), with reduction in heterogeneity (Fig 3B).

To additionally confront heterogeneity for renal function at 1 year after conversion to EVR, sensitivity analyses were done using the estimating procedures of GFR. We performed sensitivity analysis excluding 2 trials [22, 35] that reported GFR estimates based on MDRD and a similar effect to the original meta analysis was observed (MD, 5.18; 95% CI, 2.07–8.30; I^2^, 56%). We performed additional sensitivity analysis excluding 2 trials [19, 24] that did not specify just which methods used to estimate GFR and a similar effect to the original meta analysis was observed (MD, 6.30; 95% CI, 3.06–9.53; I^2^, 42%). Further, meta-regression analyses taking into consideration the baseline GFR calculations revealed that the baseline renal function did not have a substantial impact on the difference in renal function between EVR and CNI groups 1 year posttransplantation.

#### Acute rejection

**Fig 4:**
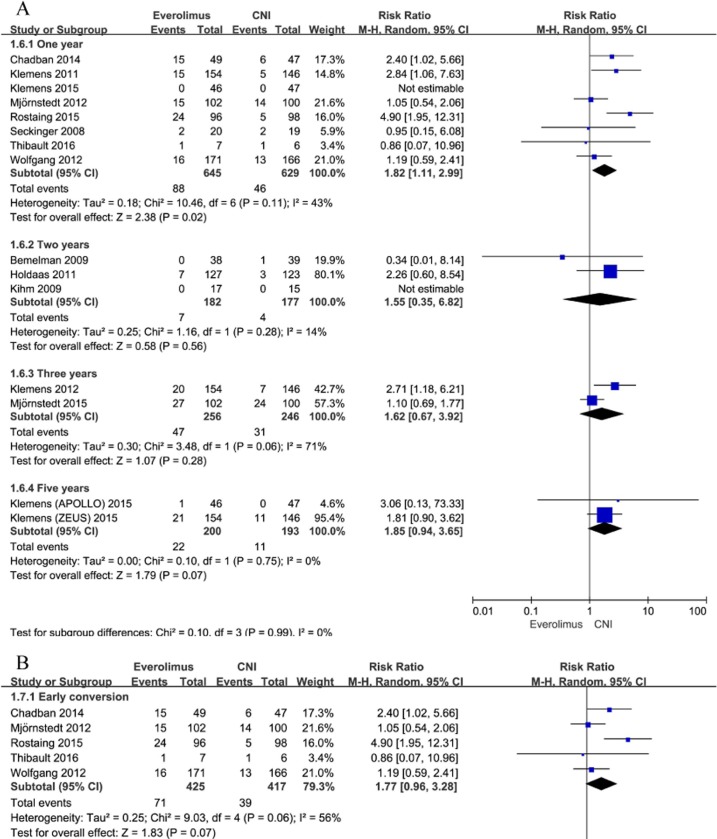
A. Everolimus versus calcineurin inhibitor; any rejection up to 5 years after transplantation stratified by study duration. B. Everolimus versus calcineurin inhibitor; any rejection up to 1 year after transplantation stratified by time to conversion.

Every one of the included studies added to the meta-analysis examining the link between conversion from CNI to EVR and acute renal allograft rejection. Every one of the trials implemented the description of biopsy-proven acute rejection (BPAR), excluding the trial by Seckinger et al [35]. In contrast to CNI maintenance, conversion to EVR was linked with a greater risk of documented acute rejection 1 year posttransplantation (RR, 1.82; 95% CI, 1.11–2.99; I^2^, 43%; Fig 4A). Analysis based on a definition of BPAR showed similar findings (RR, 1.91; 95% CI, 1.12–3.25; I^2^, 50% for patients converted to EVR). However, patients converted to EVR had a similar risk of acute rejection compared with the patients remaining on CNI up to 2, 3, and 5 years after transplantation [(RR, 1.55; 95% CI, 0.35–6.82; I^2^, 14%), (RR, 1.62; 95% CI, 0.67–3.92; I^2^, 71%), and (RR, 1.85; 95% CI, 0.94–3.65; I^2^, 0%); Fig 4A]. Moreover, we performed subgroup analysis between early conversion (between 0~3 months) and late conversion (> 3 months), and the subgroup analysis showed that early conversion to EVR compared to CNI maintenance was associated with a significantly higher risk of acute rejection up to 1 year after transplantation. In contrast, late conversion to EVR compared to CNI maintenance had a higher risk of acute rejection than CNI maintenance and it did not reach statistical significance (Fig 4B). In total, subgroup analyses did not show statistical heterogeneity among the 2 subgroups (I^2^ = 0%, p = 0.59).

#### Renal allograft loss and mortality

**Fig 5:**
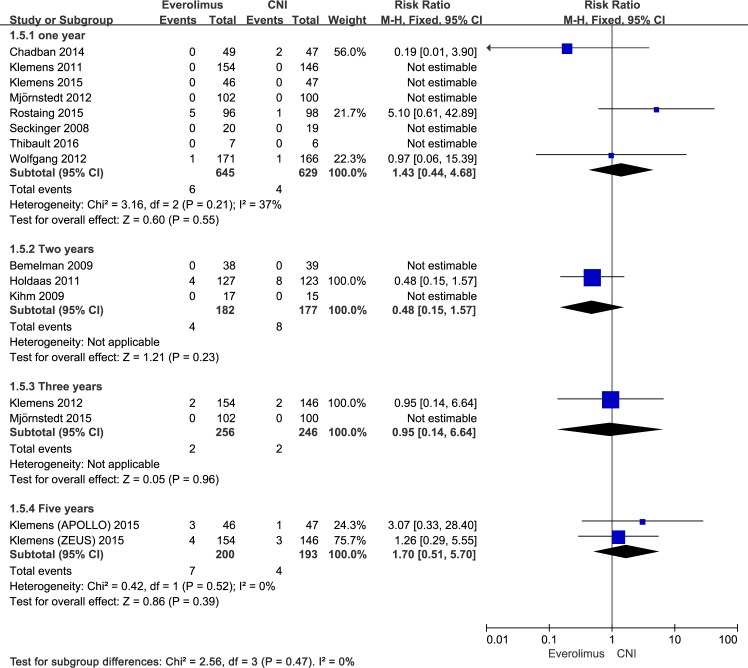
Everolimus versus calcineurin inhibitor; graft loss within 5 years after transplantation stratified by study duration.

There were 3 trials [23, 28, 31] that added to the meta-analysis examining the link between conversion from CNI to EVR and renal allograft loss while in the 5 other trials [22, 27, 30, 33, 35], no renal allografts were lost up to the first year posttransplantation. Patients converted to EVR had a similar risk of graft loss compared with the patients remaining on CNI (RR, 1.43; 95% CI, 0.44–4.68; I^2^, 37%; Fig 5). Overall, 6 (0.93%) patients in the EVR group and 4 (0.93%) patients in the CNI group lost their graft within the first year after transplantation. There was no significant difference for long-term graft loss as well.

**Fig 6:**
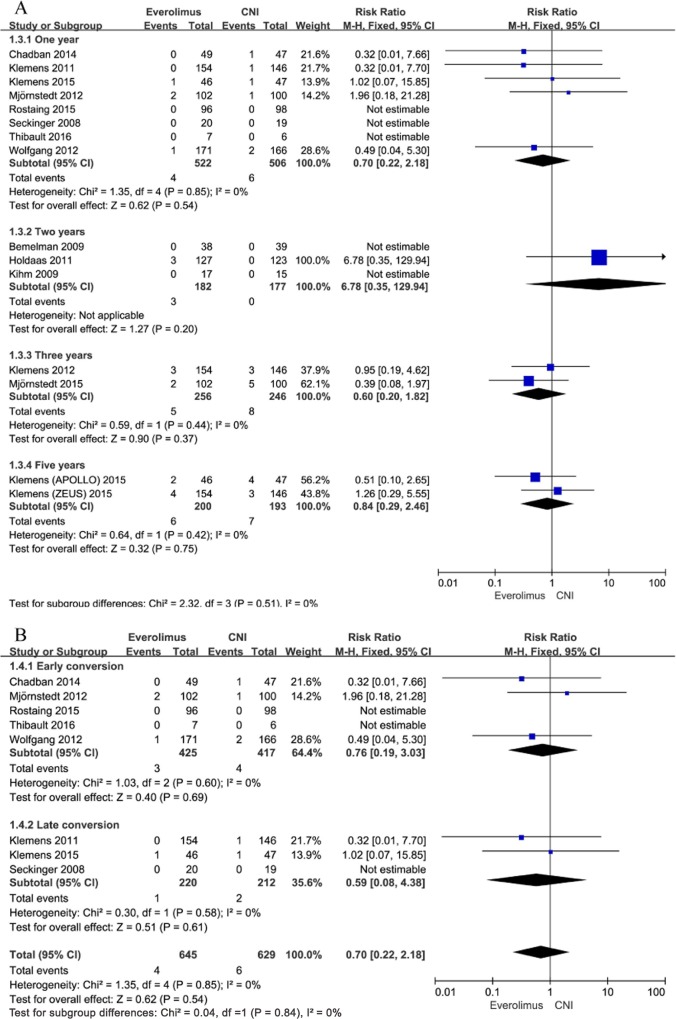
A. Everolimus versus calcineurin inhibitor; mortality up to 1–5 years after transplantation stratified by study duration. B. Everolimus versus calcineurin inhibitor; mortality up to 1 year after transplantation stratified by time to conversion.

Mortality up to 1 year posttransplantation was reported in 8 studies. Altogether, 4 (0.62%) patients in the EVR group and 6 (0.95%) patients in the CNI group died within the first year after transplantation. There were no distinctions in mortality among patients who converted to EVR and those who continued on CNI (RR, 0.70; 95% CI, 0.22–2.18; I^2^, 0%; Fig 6A). Risk ratios (RRs) and heterogeneity were alike when studies were stratified based on the time of conversion to EVR (Fig 6B). There was no significant difference for long-term mortality as well.

#### Treatment failure

Five studies [27, 28, 30, 31, 33] reported treatment failure 1 year posttransplantation. Overall, 64 (18.19%) patients in the EVR group and 29 (8.43%) patients in the CNI group failed within the first year after transplantation. There were no differences in treatment failure between patients converted to EVR and those remaining on CNI (RR, 1.74; 95% CI, 0.79–3.86; I^2^, 67%).

#### Adverse events

**Fig 7:**
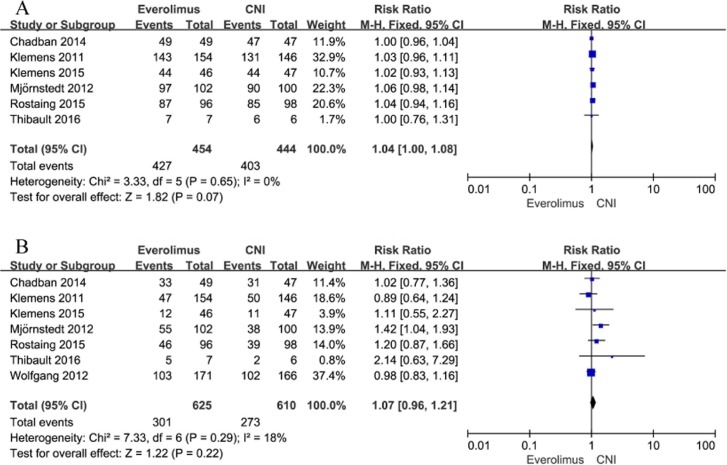
A. Everolimus versus calcineurin inhibitor; any adverse events up to 1 year after transplantation; B. Everolimus versus calcineurin inhibitor; any serious adverse events up to 1 year after transplantation.

Every one of the trials involved in the meta-analysis documented adverse events (AEs), although there were differences between studies in the nature and incidence of the reported adverse events. The main AEs involved in infection, blood and lymphatic system disorders, gastrointestinal disorders, metabolism and nutrition disorders, and the body as a whole-general disorders. Overall, 427 (94.05%) patients in the EVR group and 403 (90.77%) patients in the CNI group suffered 1 or more AEs within the first year after transplantation. There were no differences in the incidence of AEs or serious AEs between patients converted to EVR and those remaining on CNI [(RR, 1.04; 95% CI, 1.00–1.08; I^2^, 0%), and (RR, 1.07; 95% CI, 0.96–1.21; I^2^, 18%); Fig 7A and 7B]. However, the risk of study discontinuation due to AEs up to 1 year after transplantation was greater in patients converted to EVR than in patients remaining on CNI (RR, 2.63; 95% CI, 1.13–6.15; I^2^, 69%).

**Fig 8:**
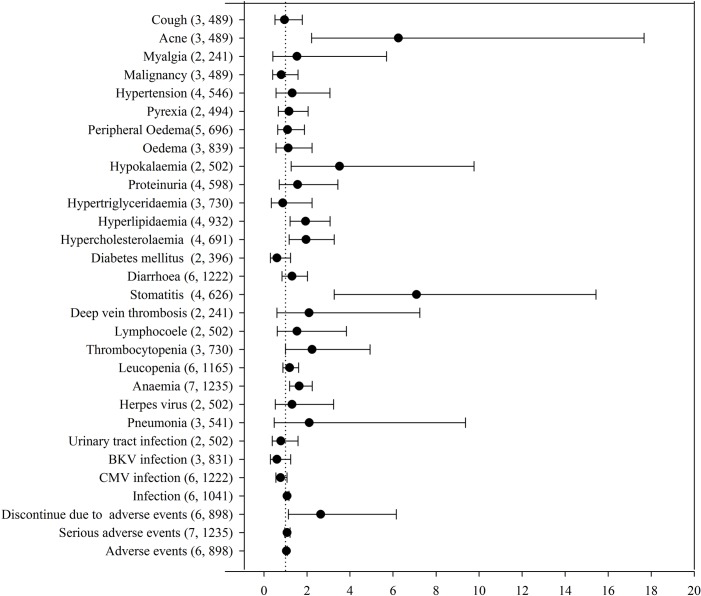
Pooled risk ratio estimates and 95% confidence intervals for adverse events up to 1 year after transplantation.

Reported adverse events, along with RR estimates and 95% CI up to 1 year after transplantation, are summarized in Fig 8 and S2 Table. Compared with patients on CNI continuation, those converted to EVR had a substantially higher risk of anemia, hyperlipidemia, hypercholesterolemia, hypokalemia, proteinuria, stomatitis, mouth ulceration, acne and a nonstatistically significant trend toward a higher risk of thrombocytopenia. Nevertheless, patients converted to EVR had a lower risk of cytomegalovirus, BK virus, and urinary tract infection, diabetes mellitus, hypertriglyceridemia, malignancy, and cough; however, it did not reach statistical significance (Table 2).

**Table 2:** Incidence of the most common adverse events up to 1 year after transplant.

Adverse events	Incidence of adverse events (%)		p-Value
	Everolimus	CNI	
anemia	13.92	8.52	0.004
hyperlipidemia	10.15	5.23	0.006
hypercholesterolaemia	11.11	5.59	0.01
hypokalaemia	6.25	1.63	0.02
proteinuria	8.55	3.53	0.007
stomatitis	18.04	1.94	<0.00001
mouth ulceration	15.38	0.75	<0.00001
acne	10.25	1.63	0.0005
thrombocytopenia	5.12	2.23	0.29
cytomegalovirus	8.90	11.59	0.11
BK virus	2.85	4.88	0.14
urinary tract infection	21.48	25.61	0.27
diabetes mellitus	4.92	8.29	0.16
hypertriglyceridaemia	2.16	2.51	0.77
malignancy	5.62	7.08	0.51
cough	6.97	7.35	0.90

As for long-term safety, we did not perform meta-analysis because there were too few trials within the same duration. The occurrence of AEs and serious AEs was similar in the EVR group versus the CNI group. The most recurrent AEs (> 10%) were anemia, diarrhea, peripheral edema, proteinuria, mouth ulceration, pyrexia, urinary tract infection, upper respiratory tract infection, increased serum creatinine, cough, rash, and acne; the first 5 were substantially elevated in the EVR group than the CNI group (p < 0.05). In addition, leukocyte and platelet counts were decreased and liver enzymes (aspartate aminotransferase and alanine aminotransferase) and lipids (total cholesterol and triglycerides) were substantially elevated in the EVR group versus the CNI group (p < 0.05). No AEs were documented less commonly in the EVR group with a statistical difference.

## Discussion

The findings from this meta-analysis establish that conversion from CNI to EVR-based maintenance immunosuppression after kidney transplantation is associated with an increased eGFR (about 10%, p < 0.01) up to 5 years after transplantation. There were no substantial differences in efficacy results between the 2 groups except that recipients who converted to EVR had a higher risk of acute graft rejection up to 1 year after transplantation. In terms of safety and tolerability, results were subordinate in the EVR group in contrast to the CNI group.

In renal transplantation, chronic allograft nephropathy (CAN) continues to be the primary reason for graft loss[37, 38]. CAN is characterized by a gradual decline of renal function with interstitial fibrosis and tubular atrophy, which causes proteinuria, high blood pressure, and a gradual escalation in serum creatinine. GFR is the most commonly utilized marker for the decline in renal function. Further, scientists have proposed that early low-grade proteinuria could offer a different marker to GFR for future risk [39–41]. Two previous meta-analysis [14, 42] evaluated mTORi for primary immunosuppression of kidney transplant recipients and concluded that mTORi showed significantly lower serum creatinine and more improved eGFR than those treated with a CNI. In the present meta-analysis, every one of the RCTs were available for analysis on renal function with GFR as the main endpoint. Our analysis conducted that eGFR was significantly elevated in the EVR group versus the CNI controls: a mean difference of 5.36mL/min per 1.73m^2^ (p = 0.0005) at 1 year and 6.50 mL/min per 1.73m^2^ (p = 0.002) 5 years after transplantation. Given the observed marked heterogeneity (I^2^, 51%) for trials reporting on the effect of EVR conversion on renal function at 1 year after transplantation, caution is required with respect to the magnitude of the overall estimate for this outcome. Subgroup and sensitivity analyses reinforced the overall conclusion that conversion to EVR was associated with improved eGFR and eliminated heterogeneity as well. Nevertheless, those patients who converted to EVR had a substantially greater risk of proteinuria (8.55% vs 3.53%, p = 0.007) at 1 year after the transplant and 2 patients developed proteinuria >1,000 mg/day post-conversion to everolimus. Offset against the insistent eGFR advantage of conversion to everolimus, this risk would seem to be allowable but because patients with elevated urine protein (e.g.> 150 or > 500 g/day) at the time of conversion seem to be more susceptible to gradual proteinuria after conversion to EVR [43], the transition should be avoided or done with care.

It has been reported that conversion from CNI to mTORi increases the risk of acute rejection [16, 22]. The present meta-analysis showed that conversion to EVR is associated with a higher risk of acute rejection at 1 year after transplantation. However, there was a similar risk of long-term acute rejection (> 1 year). Since the risk for acute rejection is highest in the first 3 months after transplantation [3], we did subgroup analysis between early conversion (between 0 and 3 months) and late conversion (> 3 months). The subgroup analysis showed that early conversion to EVR compared to CNI maintenance was associated with a significantly higher risk of acute rejection up to 1 year after transplantation; in contrast, although late conversion had a higher risk of acute rejection, it did not reach statistical significance. The meta-analysis showed that late versus early conversion to EVR was associated with a trend toward better renal function and a lower risk of acute rejection at 1 year. According to the evidence, late transition may be the better advice. In the 8 trials, only 1 trial abruptly discontinued CNI, so the present analysis did not show a difference in acute rejection between the trials evaluating abrupt and tapered discontinuation of CNI. However, experience in liver transplantation suggests that tapered discontinuation is preferable, especially when EVR conversion is introduced within the first few months of transplantation [44].

Mammalian target of rapamycin inhibitors are associated with a number of well-described side effects [4, 13, 18] that may limit the ability of patients to tolerate them. Our analysis confirmed this, indicating that the risk of study discontinuation due to AEs after EVR conversion was 2.63 times that of the patients maintained on CNIs. Overall, there was a substantial risk of adverse events after conversion to EVR, and this represents a significant barrier for its utility in preserving renal function after kidney transplantation. It was reported that EVR may lead an increased risk of bone marrow suppression [42]. Our analysis showed that conversion to EVR had a significantly higher risk of anemia, and a higher risk of thrombocytopenia, and leucopenia with no significant difference. Therefore, the real harm of EVR-induced thrombocytopenia and leukopenia is unclear from these trials. In addition, our analyses showed that conversion to EVR is associated with an increased risk of hyperlipidemia and hypercholesterolemia, although the requirements for a new statin therapy was not different to patients maintained on CNI. Conversion to EVR significantly increased the risk of stomatitis by 9.30 times (p < 0.00001) and mouth ulceration by 20.51 times (p < 0.00001) than CNI, but the rate of infection was similar to that of patients on CNI maintenance. A significant drawback of treatment with mTORi is the development of proteinuria which may reach the nephrotic range, especially after exposure to high everolimus concentrations [45]; our analysis confirmed the association between EVR and proteinuria, although the reported proteinuria levels were usually mild or less often moderate. We were unable to confirm any benefit in reduced malignancy in these 11 randomized trials, although this has been suggested by other data [46, 47]. The main limitation of this review is that of the contributory trials. Additionally, where graft-focused outcomes were well reported, many of the potentially informative patient-focused adverse outcomes were not reported, not defined, or inconsistently reported.

A longitudinal cohort study included 9,353 kidney transplant recipients showed that mTORi use was associated with a higher risk of all-cause mortality but not allograft loss [48]. The majority of RCTs included in our meta-analysis were not powered to detect a difference in graft or patient survivals, and the true effect of EVR conversion on graft and patient outcomes is still to some extent uncertain. This is especially the case for long-term outcomes, although there are 6 trials included in the meta-analysis reported outcomes beyond 1 year, they scatter in different time periods. As to long-term safety, there are too few studies to perform a meta-analysis. This review focuses on the requirement for follow-up of clinical studies in transplantation to go well beyond the immediate term to 5 years and beyond. This has been shown in renal transplantation in which a coordinated effort between the researchers and a registry of long-term patient follow-up of up to 15 years posttransplantation [49].

There are further possible restrictions in the current meta-analysis. First, we did not incorporate any unpublished evidence, so there may be publication bias in the meta-analysis. Second, even though randomized evidence is safeguarded from selection bias, performance and detection biases could be possible confounders. An approach toward addressing this would be to exempt open-label trials. Ultimately, the majority of the incorporated trials were, by necessity, open-label trials, and so trial exclusion was not a viable choice. Lastly, a group of study parameters could possibly impede our study outcomes. The variation in baseline renal function between studies, and the unmistakable patient characteristics inside separate studies may have added to the noted heterogeneity.

## Conclusion

The currently available randomized evidence indicates that conversion from CNI to EVR after kidney transplantation is associated with short and long-term improvements in GFR in a number of studies, but these findings must be balanced against the greater rates of proteinuria, discontinuation due to AEs, and higher incidences of acute rejection up to 1 year posttransplant with the use of EVR as compared with CNI. Moreover, late conversion to EVR might be associated with a trend toward better renal function and a lower risk of acute rejection up to 1 year posttransplantion than early conversion. Additionally, in deciding the optimal immunosuppression strategy for their patients, clinicians should be alert to the increased risk of bone marrow suppression, dyslipidemia, mouth ulceration, and stomatitis after conversion to EVR which, however, has no effect on graft and patient survival.

## Supporting Information

S1 Fig Risk of bias summary: each risk of bias item for each included study. (TIF)

S2 Fig Funnel plot of pooled efficacy and safety of everolimus comparing with calcineurin inhibitor. (TIF)

S1 Table Literature search algorithm. (DOCX)

S2 Table Effect estimate of all outcomes. (DOCX)

S3 Table PRISMA checklist. (DOC)

S1 Text Abbreviations page. (DOCX)

S2 Text Flow diagram. (DOC)
