# Supplementary figures and images for: Efficacy and Safety of Everolimus for Maintenance Immunosuppression of Kidney Transplantation: A Meta-Analysis of Randomized Controlled Trials
Source: PLoS One. 2017 Jan 20;12(1):e0170246. doi: 10.1371/journal.pone.0170246 (PMC5249216; doi:10.1371/journal.pone.0170246)

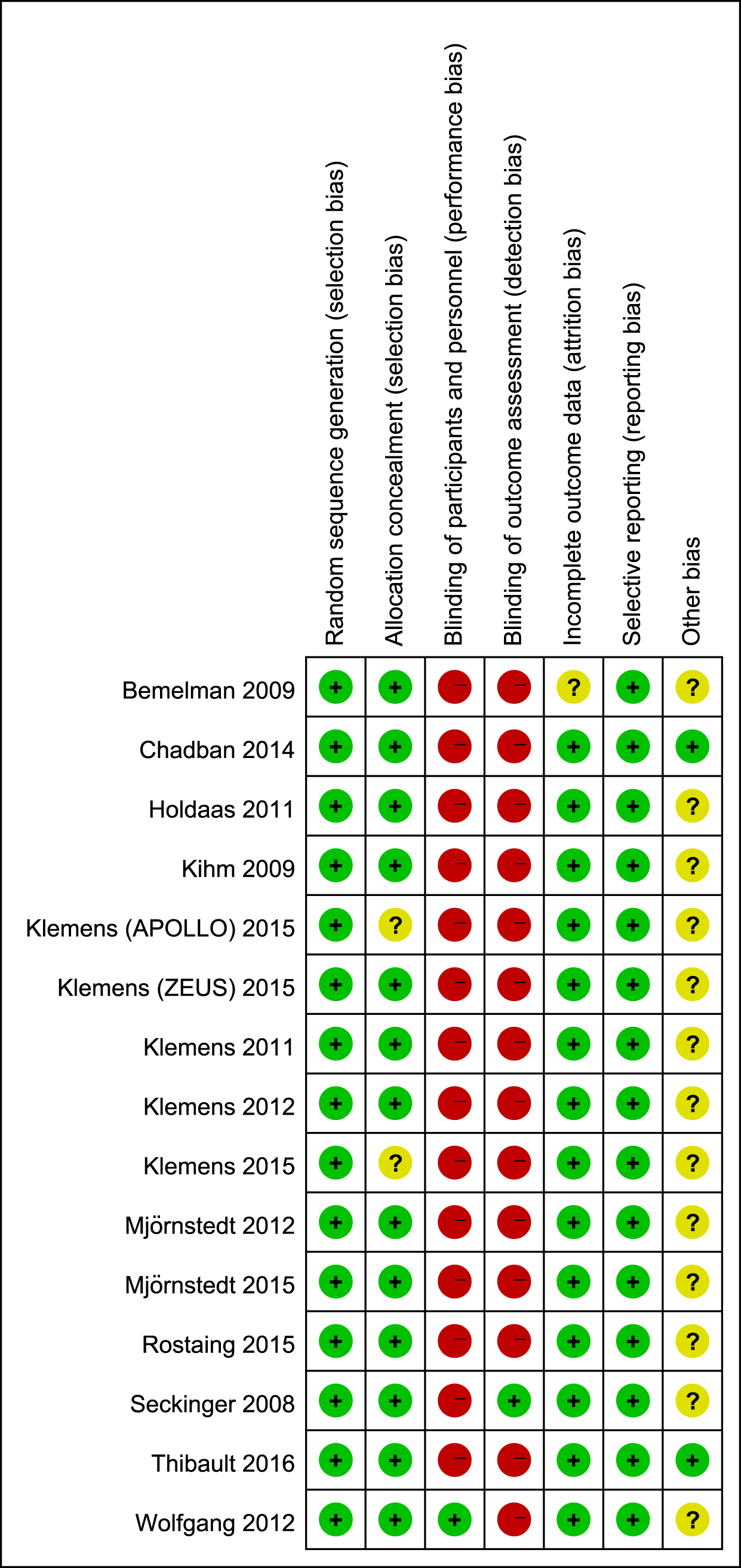

Supplement: S1 Fig — (TIF) [file pone.0170246.s001.tif]

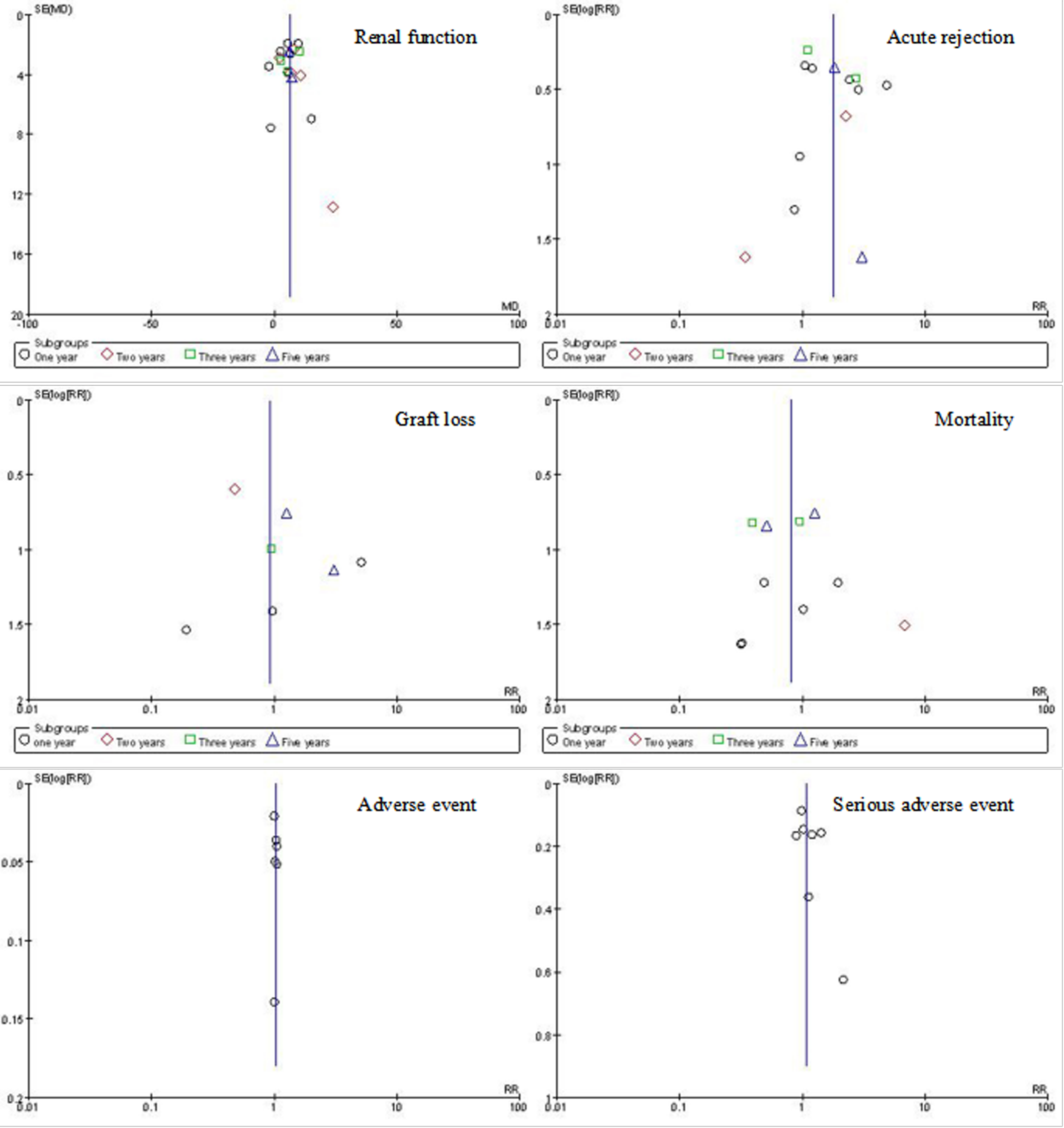

Supplement: S2 Fig — (TIF) [file pone.0170246.s002.tif]
