# Supplementary material for: Efficacy and Safety of Everolimus for Maintenance Immunosuppression of Kidney Transplantation: A Meta-Analysis of Randomized Controlled Trials
Source: PLoS One. 2017 Jan 20;12(1):e0170246. doi: 10.1371/journal.pone.0170246 (PMC5249216; doi:10.1371/journal.pone.0170246)
Supplement: S1 Table — (DOCX) [file pone.0170246.s003.docx]

**S1Table . Literature search algorithm**

**MEDLINE (PubMed)**

| #1 | (kidney transplantation[MeSH] OR( (renal [tiab] OR kidney[tiab]) AND (transplant[tiab] OR transplantation[tiab] OR graft[tiab] OR graf*[tiab] OR transplant*[tiab]))) | 121519 |
| --- | --- | --- |
| #2 | ((Everolimus[MeSH]OR rapamycin derivative[tiab] OR rapamune[tiab] OR mtor inhibitor[tiab] OR mTORC1 inhibitor[tiab] OR TOR inhibitor[tiab] OR mammalian target of rapamycin[tiab] OR rapamycin*[tiab] OR everolimus[tiab] OR RAD[tiab] OR SDZ[tiab] OR RAD001[tiab]) AND (calcineurin inhibitor[tiab] OR cyclosporine[tiab] OR ciclosporin*[tiab] OR neoral*[tiab] OR sandimmun*[tiab] OR tacrolimus[tiab] OR FK506[tiab] OR prograf[tiab] OR advagraf[tiab])) | 2448 |
| #3 | #1 AND #2 AND (Humans[species]) AND (2000/01/01-2016/03/31[Publication date]) | 673 |

**EMBASE (Ovid)**

| #1 | (‘kidney transplantation’/exp OR ( (renal:ti,ab OR kidney:ti,ab) AND (transplant:ti,ab OR transplantation:ti,ab OR graft:ti,ab OR graf*:ti,ab OR transplant*:ti,ab))) | 177010 |
| --- | --- | --- |
| #2 | ((Everolimus/exp OR ‘rapamycin derivative’:ti,ab OR rapamune:ti,ab OR ‘mtor inhibitor’:ti,ab OR ‘mTORC1 inhibitor’:ti,ab OR ‘TOR inhibitor’:ti,ab OR ‘mammalian target of rapamycin’:ti,ab OR rapamycin*:ti,ab OR everolimus:ti,ab OR RAD:ti,ab OR SDZ:ti,ab OR RAD001:ti,ab) AND (calcineurin inhibitor:ti,ab OR cyclosporine:ti,ab OR ciclosporin*:ti,ab OR neoral*:ti,ab OR sandimmun*:ti,ab OR tacrolimus:ti,ab OR FK506:ti,ab OR prograf:ti,ab OR advagraf:ti,ab) | 4420 |
| #3 | #1 AND #2 AND ([Human]/lim) AND ([2000-2016]/py) | 1801 |

**Cochrane Library**

| #1 | (‘kidney transplantation’/exp OR ((renal:ti,ab,kw OR kidney:ti,ab,kw) AND (transplant:ti,ab,kw OR transplantation:ti,ab,kw OR graft:ti,ab,kw OR graf*:ti,ab,kw OR transplant*:ti,ab,kw))) | 177010 |
| --- | --- | --- |
| #2 | ((Everolimus/exp OR ‘rapamycin derivative’:ti,ab,kw OR rapamune:ti,ab,kw OR ‘mtor inhibitor’:ti,ab,kw OR ‘mTORC1 inhibitor’:ti,ab,kw OR ‘TOR inhibitor’:ti,ab,kw OR ‘mammalian target of rapamycin’:ti,ab,kw OR rapamycin*:ti,ab,kw OR everolimus:ti,ab,kw OR RAD:ti,ab,kw OR SDZ:ti,ab,kw OR RAD001:ti,ab,kw) AND (calcineurin inhibitor:ti,ab,kw OR cyclosporine:ti,ab,kw OR ciclosporin*:ti,ab,kw OR neoral*:ti,ab,kw OR sandimmun*:ti,ab,kw OR tacrolimus:ti,ab,kw OR FK506:ti,ab,kw OR prograf:ti,ab,kw OR advagraf:ti,ab,kw) | 4420 |
| #3 | #1 AND #2 | 643 |
