# Supplementary material for: Efficacy and Safety of Everolimus for Maintenance Immunosuppression of Kidney Transplantation: A Meta-Analysis of Randomized Controlled Trials
Source: PLoS One. 2017 Jan 20;12(1):e0170246. doi: 10.1371/journal.pone.0170246 (PMC5249216; doi:10.1371/journal.pone.0170246)
Supplement: S2 Table — (DOCX) [file pone.0170246.s004.docx]

Table S2. Effect estimate of all outcomes

| Outcome or subgroup | Studies | Participants | Statistical Method | Effect Estimate |
| --- | --- | --- | --- | --- |
| 1.1 renal function | 15 |  | Mean Difference (IV, Random, 95% CI) | Subtotals only |
| 1.1.1 One year | 8 | 1237 | Mean Difference (IV, Random, 95% CI) | 5.36 [2.32, 8.39] |
| 1.1.2 Two years | 5 | 652 | Mean Difference (IV, Random, 95% CI) | 6.91 [3.04, 10.79] |
| 1.1.3 Three years | 3 | 483 | Mean Difference (IV, Random, 95% CI) | 6.65 [1.61, 11.70] |
| 1.1.4 Five years | 2 | 285 | Mean Difference (IV, Random, 95% CI) | 6.50 [2.38, 10.63] |
| 1.2 renal function (1year) | 8 | 1240 | Mean Difference (IV, Random, 95% CI) | 5.36 [2.32, 8.40] |
| 1.2.1 Early conversion | 5 | 842 | Mean Difference (IV, Random, 95% CI) | 3.65 [0.70, 6.60] |
| 1.2.2 Late conversion | 3 | 398 | Mean Difference (IV, Random, 95% CI) | 9.42 [6.13, 12.70] |
| 1.3 Mortality | 12 |  | Risk Ratio (M-H, Fixed, 95% CI) | Subtotals only |
| 1.3.1 One year | 5 | 1028 | Risk Ratio (M-H, Fixed, 95% CI) | 0.70 [0.22, 2.18] |
| 1.3.2 Two years | 3 | 359 | Risk Ratio (M-H, Fixed, 95% CI) | 6.78 [0.35, 129.94] |
| 1.3.3 Three years | 2 | 502 | Risk Ratio (M-H, Fixed, 95% CI) | 0.60 [0.20, 1.82] |
| 1.3.4 Five years | 2 | 393 | Risk Ratio (M-H, Fixed, 95% CI) | 0.84 [0.28, 2.54] |
| 1.4 Mortality (1year) | 8 | 1274 | Risk Ratio (M-H, Fixed, 95% CI) | 0.70 [0.22, 2.18] |
| 1.4.1 Early conversion | 5 | 842 | Risk Ratio (M-H, Fixed, 95% CI) | 0.76 [0.19, 3.03] |
| 1.4.2 Late conversion | 3 | 432 | Risk Ratio (M-H, Fixed, 95% CI) | 0.59 [0.08, 4.38] |
| 1.5 Graft loss | 15 |  | Risk Ratio (M-H, Fixed, 95% CI) | Subtotals only |
| 1.5.1 one year | 8 | 1274 | Risk Ratio (M-H, Fixed, 95% CI) | 1.43 [0.44, 4.68] |
| 1.5.2 Two years | 3 | 359 | Risk Ratio (M-H, Fixed, 95% CI) | 0.48 [0.15, 1.57] |
| 1.5.3 Three years | 2 | 502 | Risk Ratio (M-H, Fixed, 95% CI) | 0.95 [0.14, 6.64] |
| 1.5.4 Five years | 2 | 393 | Risk Ratio (M-H, Fixed, 95% CI) | 1.70 [0.51, 5.70] |
| 1.6 Acute rejection | 15 |  | Risk Ratio (M-H, Random, 95% CI) | Subtotals only |
| 1.6.1 One year | 8 | 1274 | Risk Ratio (M-H, Random, 95% CI) | 1.82 [1.11, 2.99] |
| 1.6.2 Two years | 3 | 359 | Risk Ratio (M-H, Random, 95% CI) | 1.55 [0.35, 6.82] |
| 1.6.3 Three years | 2 | 502 | Risk Ratio (M-H, Random, 95% CI) | 1.62 [0.67, 3.92] |
| 1.6.4 Five years | 2 | 393 | Risk Ratio (M-H, Random, 95% CI) | 1.85 [0.94, 3.65] |
| 1.7 acute rejection (1year) | 7 | 1181 | Risk Ratio (M-H, Random, 95% CI) | 1.82 [1.11, 2.99] |
| 1.7.1 Early conversion | 5 | 842 | Risk Ratio (M-H, Random, 95% CI) | 1.77 [0.96, 3.28] |
| 1.7.2 Late conversion | 2 | 339 | Risk Ratio (M-H, Random, 95% CI) | 2.20 [0.89, 5.48] |
| 1.8 Treatment failure | 5 | 696 | Risk Ratio (M-H, Random, 95% CI) | 1.74 [0.79, 3.86] |
| 1.9 ADR | 6 | 898 | Risk Ratio (M-H, Fixed, 95% CI) | 1.04 [1.00, 1.08] |
| 1.10 Serious ADR | 7 | 1235 | Risk Ratio (M-H, Fixed, 95% CI) | 1.07 [0.96, 1.21] |
| 1.11 Discontinue due to ADR | 6 | 898 | Risk Ratio (M-H, Random, 95% CI) | 2.63 [1.13, 6.15] |
| 1.12 Infection | 8 |  | Risk Ratio (M-H, Fixed, 95% CI) | Subtotals only |
| 1.12.1 Any infection | 6 | 1041 | Risk Ratio (M-H, Fixed, 95% CI) | 1.09 [1.00, 1.18] |
| 1.12.2 Cytomegalovirus | 6 | 1222 | Risk Ratio (M-H, Fixed, 95% CI) | 0.76 [0.55, 1.06] |
| 1.12.3 BK virus | 3 | 831 | Risk Ratio (M-H, Fixed, 95% CI) | 0.59 [0.29, 1.18] |
| 1.12.4 Pneumonia | 3 | 541 | Risk Ratio (M-H, Fixed, 95% CI) | 1.88 [0.91, 3.88] |
| 1.12.5 Herpes virus | 2 | 502 | Risk Ratio (M-H, Fixed, 95% CI) | 1.32 [0.62, 2.81] |
| 1.12.6 Urinary tract infection | 2 | 502 | Risk Ratio (M-H, Fixed, 95% CI) | 0.84 [0.61, 1.15] |
| 1.13 Blood and lymphatic system disorders | 8 |  | Risk Ratio (M-H, Random, 95% CI) | Subtotals only |
| 1.13.1 Anaemia | 7 | 1235 | Risk Ratio (M-H, Random, 95% CI) | 1.60 [1.16, 2.20] |
| 1.13.2 Leucopenia | 6 | 1165 | Risk Ratio (M-H, Random, 95% CI) | 1.15 [0.74, 1.79] |
| 1.13.3 Thrombocytopenia | 3 | 730 | Risk Ratio (M-H, Random, 95% CI) | 2.37 [0.48, 11.77] |
| 1.13.4 Lymphocoele | 2 | 502 | Risk Ratio (M-H, Random, 95% CI) | 1.54 [0.61, 3.85] |
| 1.13.5 Deep vein thrombosis | 2 | 241 | Risk Ratio (M-H, Random, 95% CI) | 2.08 [0.60, 7.24] |
| 1.14 Gastrointestinal disorders | 7 |  | Risk Ratio (M-H, Random, 95% CI) | Subtotals only |
| 1.14.1 Stomatitis | 4 | 626 | Risk Ratio (M-H, Random, 95% CI) | 7.09 [3.26, 15.43] |
| 1.14.2 Diarrhoea | 6 | 1222 | Risk Ratio (M-H, Random, 95% CI) | 1.30 [0.83, 2.02] |
| 1.14.3 Mouth ulceration | 2 | 539 | Risk Ratio (M-H, Random, 95% CI) | 19.07 [4.64, 78.34] |
| 1.15 Metabolism and nutrition disorders | 5 |  | Risk Ratio (M-H, Fixed, 95% CI) | Subtotals only |
| 1.15.1 Diabetes mellitus | 2 | 396 | Risk Ratio (M-H, Fixed, 95% CI) | 0.60 [0.29, 1.23] |
| 1.15.2 Hypercholesterolaemia | 4 | 691 | Risk Ratio (M-H, Fixed, 95% CI) | 1.95 [1.17, 3.26] |
| 1.15.3 Hyperlipidaemia | 4 | 932 | Risk Ratio (M-H, Fixed, 95% CI) | 1.93 [1.21, 3.07] |
| 1.15.4 Hypertriglyceridaemia | 3 | 730 | Risk Ratio (M-H, Fixed, 95% CI) | 0.87 [0.34, 2.23] |
| 1.15.5 Proteinuria | 4 | 691 | Risk Ratio (M-H, Fixed, 95% CI) | 2.39 [1.27, 4.49] |
| 1.15.6 Hypokalaemia | 2 | 502 | Risk Ratio (M-H, Fixed, 95% CI) | 3.51 [1.26, 9.77] |
| 1.16 Body as a whole-general disorders | 7 |  | Risk Ratio (M-H, Random, 95% CI) | Subtotals only |
| 1.16.1 Oedema | 3 | 839 | Risk Ratio (M-H, Random, 95% CI) | 1.12 [0.56, 2.23] |
| 1.16.2 Peripheral Oedema | 5 | 696 | Risk Ratio (M-H, Random, 95% CI) | 1.09 [0.63, 1.88] |
| 1.16.3 Pyrexia | 2 | 494 | Risk Ratio (M-H, Random, 95% CI) | 1.16 [0.66, 2.05] |
| 1.17 Other disorders | 7 |  | Risk Ratio (M-H, Random, 95% CI) | Subtotals only |
| 1.17.1 Hypertension | 4 | 546 | Risk Ratio (M-H, Random, 95% CI) | 1.29 [0.53, 3.14] |
| 1.17.2 Malignancy | 3 | 489 | Risk Ratio (M-H, Random, 95% CI) | 0.79 [0.40, 1.58] |
| 1.17.3 Myalgia | 2 | 241 | Risk Ratio (M-H, Random, 95% CI) | 1.40 [0.23, 8.38] |
| 1.17.4 Acne | 3 | 489 | Risk Ratio (M-H, Random, 95% CI) | 6.25 [2.21, 17.67] |
| 1.17.5 Cough | 3 | 489 | Risk Ratio (M-H, Random, 95% CI) | 0.96 [0.51, 1.83] |
