# Supplementary material for: Efficacy and Safety of Everolimus for Maintenance Immunosuppression of Kidney Transplantation: A Meta-Analysis of Randomized Controlled Trials
Source: PLoS One. 2017 Jan 20;12(1):e0170246. doi: 10.1371/journal.pone.0170246 (PMC5249216; doi:10.1371/journal.pone.0170246)
Supplement: S1 Text — (DOCX) [file pone.0170246.s006.docx]

CNI, calcineurin inhibitor; mTORi, mammalian target of rapamycin inhibitor; CsA, cyclosporine A; EVR, everolimus; TaC, tacrolimus; Aza, azathioprine; MPA, mycophenolic acid; EC-MPS, enteric-coated mycophenolate sodium; RCT, randomized controlled trail; CI, confidence intervals; RR, risk ratio; MD, mean difference; SD, standard deviation; GFR, glomerular filtration rate; ITT, intention-to-treat; MDRD, modification of diet in renal disease; BPAR, biopsy-proven acute rejection; AEs, adverse effects
