# Supplementary material for: Efficacy and Safety of Everolimus for Maintenance Immunosuppression of Kidney Transplantation: A Meta-Analysis of Randomized Controlled Trials
Source: PLoS One. 2017 Jan 20;12(1):e0170246. doi: 10.1371/journal.pone.0170246 (PMC5249216; doi:10.1371/journal.pone.0170246)
Supplement: S2 Text — (DOC) [file pone.0170246.s007.doc]

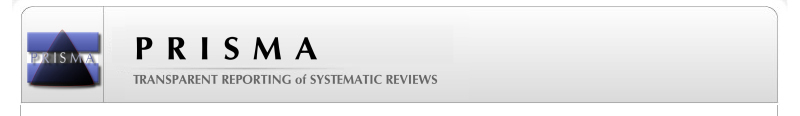
**PRISMA 2009 Flow Diagram**

**Screening**

**Included**

**Eligibility**

**Identification**

Records identified through database searching
(n =3117 )

Additional records identified through other sources
(n = 12 )

Records after duplicates removed
(n =1874 )

Records screened
(n = 1874 )

Records excluded
(n = 1807 )

Full-text articles assessed for eligibility
(n = 67 )

Full-text articles excluded, with reasons
(n =52 )

Studies included in qualitative synthesis
(n = 15 )

Studies included in quantitative synthesis (meta-analysis)
(n =15 )
